# Time scale matters: genetic analysis does not support adaptation-by-time as the mechanism for adaptive seasonal declines in kokanee reproductive life span

**DOI:** 10.1002/ece3.1214

**Published:** 2014-09-05

**Authors:** Yolanda E Morbey, Evelyn L Jensen, Michael A Russello

**Affiliations:** 1 Department of Biology, Western UniversityLondon, Ontario, N6A 5B7, Canada; 2 Department of Biology, University of British Columbia, Okanagan CampusKelowna, British Columbia, V1V 1V7, Canada

**Keywords:** Adaptation-by-time, evolutionary mechanisms, isolation-by-time, reproductive timing, seasonal declines in fitness-related traits

## Abstract

Seasonal declines of fitness-related traits are often attributed to environmental effects or individual-level decisions about reproductive timing and effort, but genetic variation may also play a role. In populations of Pacific salmon (*Oncorhynchus* spp.), seasonal declines in reproductive life span have been attributed to adaptation-by-time, in which divergent selection for different traits occurs among reproductively isolated temporal components of a population. We evaluated this hypothesis in kokanee (freshwater obligate *Oncorhynchus nerka*) by testing for temporal genetic structure in neutral and circadian-linked loci. We detected no genetic differences in presumably neutral loci among kokanee with different arrival and maturation dates within a spawning season. Similarly, we detected no temporal genetic structure in *OtsClock1b*, *Omy1009uw*, or *OmyFbxw11*, candidate loci associated with circadian function. The genetic evidence from this study and others indicates a lack of support for adaptation-by-time as an important evolutionary mechanism underlying seasonal declines in reproductive life span and a need for greater consideration of other mechanisms such as time-dependent, adaptive adjustment of reproductive effort.

## Introduction

In seasonal breeders, traits related to reproductive effort and parental care often decline with breeding date. In temperate-breeding birds, for example, later breeders may have smaller clutch sizes, slower nestling growth rates, or lower fledging masses (Brinkhof 1995; Ydenberg et al. 1995; Verhulst and Nilsson 2008). In Pacific salmon in the genus *Oncorhynchus*, fish arriving later for spawning often have short reproductive life spans (RLS), resulting in less time available for reproductive activities and parental care prior to their rapid physiological decline and death. These declines in RLS are apparent over short arrival periods of 12–35 days in multiple populations and species and are quite steep (Hendry and Day 2005). Multiple evolutionary and mechanistic hypotheses have been proposed to explain seasonal declines, but no single hypothesis dominates. Based on extensive observational and experimental data, seasonal declines in fitness-related traits often can be attributed to the later arrival of poorer quality parents, seasonal declines in food availability, or an interaction between the two (Verhulst and Nilsson 2008). In addition to such direct effects on reproductive investment, parents may adjust their reproductive effort downward in response to deteriorating environmental conditions or time constraints. In magpies, for example, parents forced to renest built smaller nests and laid smaller replacement clutches with larger eggs (de Neve and Soler 2002; de Neve et al. 2004). Finally, adaptive seasonal declines in response to temporally variable selection may evolve via genetic adaptation in a process called adaptation-by-time (Hendry and Day 2005). In contrast to the other hypotheses, adaptation-by-time requires genetic constraints on breeding date (i.e., a high heritability). Our objective was to evaluate adaptation-by-time as an explanation for seasonal declines in RLS in kokanee, the freshwater obligate form of sockeye salmon (*Oncorhynchus nerka* Walbaum).

While adaptation-by-time can apply to any seasonally varying trait, seasonal declines in RLS are an obvious contender because of seasonally varying selective pressures, highly heritable breeding dates (usually >0.5), and evidence of reproductive isolation-by-time (Hendry and Day 2005). Theory indicates that seasonal declines in RLS in females are adaptive as part of a digup avoidance strategy. Whereas early-arriving females live longer to defend their territories against late-arriving conspecifics, late-arriving females face fewer intruders and instead invest their limited resources into egg production (Morbey and Ydenberg 2003; Hendry et al. 2004). In males, seasonal declines in RLS are consistent with a seasonally varying trade-off between maximizing mating opportunities via male presence versus investing in competitive ability (Morbey and Abrams 2004). Importantly, the presence of steep seasonal declines in RLS requires breeding periods of intermediate durations. Under maximum synchrony, all individuals should live for a very short time; under maximum asynchrony, all individuals should live for the maximum RLS imposed by physiological trade-offs. Given temporally variable selection pressures, reproductive isolation-by-time at the same time scale should then facilitate the divergence of RLS between early- and late-arriving fish.

Few studies of reproductive isolation-by-time consider time scales that match the time scale for seasonal declines in RLS, thus calling into question the importance of adaptation-by-time in contributing to these declines. Several studies report weak or no isolation-by-time at time scales less than 1 month (Table 2 in Hendry and Day 2005). For example, in Tustemena Lake, Alaska, sockeye salmon (anadromous *O. nerka*) entering a creek 21–25 day apart or 13–15 day apart, *F*_ST_ was 0.006 (significant) and 0.003 (nonsignificant), respectively (Woody et al. 2000). In Pick Creek, Alaska, sockeye salmon collected 29 day apart had low but limited gene flow (*m* = 0.00023) (Hendry et al. 2004). Hendry and Day (2005) detected significant isolation-by-time for Cedar River sockeye salmon over 2 months, but the pattern disappears for pairwise time differences of less than 1 month. Moreover, of these papers, seasonal declines in RLS were assessed and quantified only in Hendry et al. (2004). Further case studies would help clarify whether adaptive seasonal declines in RLS have an underlying genetic basis or whether nongenetic explanations should be given greater attention.

Our case study focuses on a population of kokanee salmon known to express seasonal declines in RLS (Morbey and Ydenberg 2003). If adaptation-by-time plays a significant role in the evolution of seasonal declines in RLS in kokanee, we predict reproductive isolation-by-time between early- and late-arriving fish based on neutral loci (Hendry and Day 2005). This was tested by analyzing variation at nine microsatellite loci, previously used in kokanee (Taylor et al. 2000; Lemay and Russello 2012). We also predicted differentiation at circadian-linked loci, because of their role in the regulation of seasonal timing. In salmon, *OtsClock1b* is one of two duplicated clock genes, and length polymorphisms of the PolyQ domain are known to vary among populations with distinct reproductive timing at different scales (O’Malley et al. 2007, 2010; O’Malley and Banks 2008), including in kokanee (Lemay and Russello 2014).

## Methods

### Sample collection

**Figure 1:**
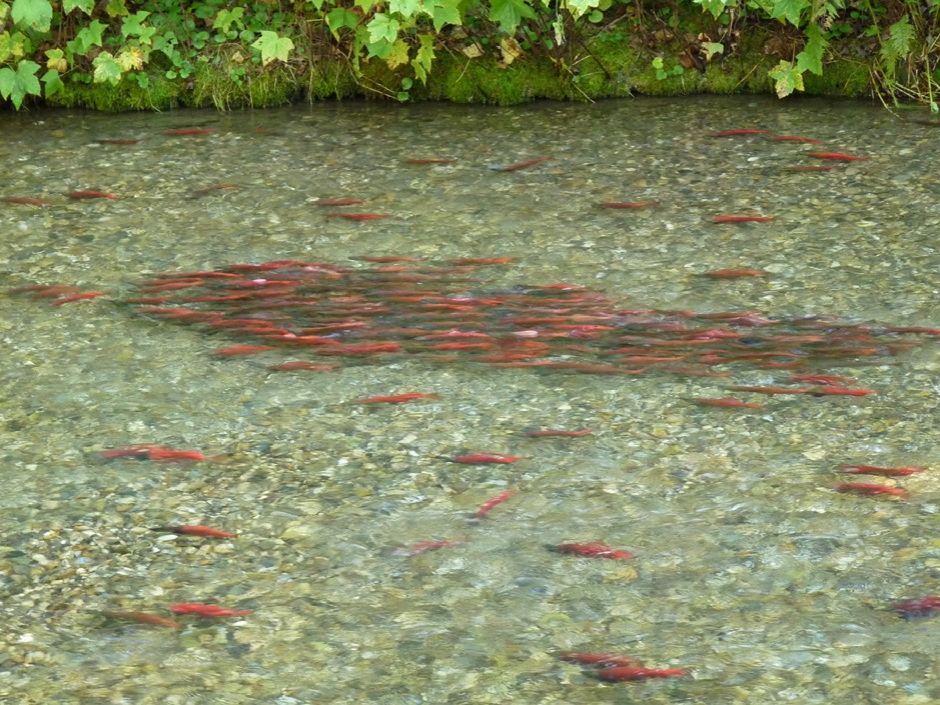
Kokanee at the Meadow Creek Spawning Channel, BC, Canada, in September, 2013. Shown are spawning pairs and a single large shoal with males and females of various maturation states. Shoals will eventually disperse as female complete sexual maturation and seek out nesting sites and as males leave to seek reproductive opportunities.

We studied kokanee (Fig. 1) at the Meadow Creek Spawning Channel at the north end of Kootenay Lake, British Columbia, Canada, in 2013. Individuals end their migration at an enumeration fence and generally wait less than a day before entering the channel via an open gate. Most spawning occurs in September, but arrival timing can vary among years. During 1990–2013, there has been a 20-day range in the first date of enumeration (M. Pearson, BC Ministry of Forests, Lands, and Natural Resource Operations, pers. comm.). In 2013, the first day of enumeration was 5 September and was the latest on record. In all years studied, maturation state at arrival varies extensively, with many females completing sexual maturation after arrival (Morbey and Guglielmo 2006; Warren and Morbey 2011). Females are considered to be sexually mature if they are very red and if loose eggs can be pushed out with gentle abdominal compression. In contrast, immature females are still silvery.

Over 18 days in September 2013, we used dip nets to capture females at the enumeration fence. A short breeding season is typical of this population, and previous research shows steep seasonal declines in the period between arrival and death over a 21-day arrival period (Morbey and Ydenberg 2003). We aimed to sample groups of females with divergent maturation dates. Thus, we collected red females early and late in the arrival period. We also collected early-arriving silver females who would have spawned late. In addition, some early-arriving silver females were transferred into a pen until sexual maturation and terminal sampling. We regularly recaptured and assessed these females until they reached sexual maturity. We euthanized all females with an overdose of clove oil. For this study, a cut piece of operculum was preserved in 95% ethanol. We also confirmed the presence or absence of loose, ovulated eggs. In the end, we collected tissue samples from 109 females among four groups: RE = red early (7–11 September; *n* = 31), RL = red late (15–22 September; *n* = 21), SE = silver early (5–8 September; *n* = 33), and SR = silver that arrived early (5–8 September) but matured in a pen (14–23 September; *n* = 24). With these groups, we were able to compare genetic variation on the basis of arrival date or maturation date. All procedures performed on live animals were approved by Western University’s Animal Use Subcommittee (AUP 2013-033).

### Genetic analysis

DNA was extracted from each sample using a NucleoSpin Tissue Kit (Macherey-Nagel) following manufacturer’s protocols. Nuclear genotypic data were collected from nine neutral microsatellite loci and three circadian-linked microsatellites or genes with fragment length polymorphisms (Table 1). All forward primers were 5′-tailed with an M13 sequence and used in combination with an M13 primer of the same sequence 5′-labeled with one of four fluorescent dyes (6-FAM, VIC, NED, PET) to facilitate automated genotyping (Schuelke 2000). Polymerase chain reactions (PCRs) were carried out on an ABI Veriti thermal cycler in 15 *μ*l reactions containing ~ 20 ng DNA, 1.25 *μ*L of 10 × PCR buffer, 1.25 *μ*L of 2 mm dNTP mix, 0.5 *μ*L of 1 *μ*m forward primer, 0.5 *μ*L of 10 *μ*m M13 fluorescent-labeled primer, 0.5 *μ*L of 10 *μ*m reverse primer, and 0.5 units of Kapa*Taq* polymerase. The PCR was carried out with an initial denaturation of 94^°^C for 2 min, followed by 20 cycles at 94^°^C for 30 sec, 60^°^C for 30 sec, and 72^°^C for 30 sec, with the annealing temperature decreasing by 0.5^°^C in each cycle to 50^°^C. The annealing temperature was maintained at 50^°^C for another 15 cycles followed by a final extension at 72^°^C for 2 min. Loci were coloaded and run on an Applied Biosystems 3130XL DNA automated sequencer. Alleles were scored using bins in the software Genemapper 4.0 (Applied Biosystems, Foster City, California, USA), and all allele calls were manually verified.

**Table 1:** Locus information and diversity indices over all genotyped individuals

	Locus	*N*_A_	*H*_o_	*H*_e_	M13 Dye	Source
Circadian linked	*OtsClock1b*	3	0.48	0.48	FAM-6	O’Malley et al. (2007)
	*Omy1009uw*	4	0.08	0.13	PET	Spies et al. (2005)
	*OmyFbxw11*	20	0.64	0.58	FAM-6	O’Malley et al. (2013)
Neutral	*Ev149*	12	0.90	0.80	PET	Koop et al. (2008)
	*Ev358*	30	0.95	0.87	NED	Koop et al. (2008)
	*Omm5058*	22	0.90	0.85	PET	Rexroad et al. (2005)
	*One102*	18	0.93	0.90	NED	Olsen et al. (1996)
	*One112*	27	0.95	0.93	NED	Olsen et al. (1996)
	*One8*	9	0.37	0.35	VIC	Scribner et al. (1996)
	*Ots100*	24	0.84	0.88	VIC	Nelson and Beacham (1999)
	*Ots103*	23	0.91	0.93	FAM-6	Nelson and Beacham (1999)
Neutral average		14.2	0.84	0.81		

*N*_A_, number of alleles; *H*_o_, observed heterozygosity; *H*_e_, expected heterozygosity.

Microsatellite loci were tested for null and false alleles using MICROCHECKER (Van Oosterhout et al. 2004). Deviation from Hardy–Weinberg equilibrium (HWE) was assessed using the Markov chain Monte Carlo (MCMC) approximation of Fisher’s exact test using 1000 batches with 1000 iterations (Guo and Thompson 1992), and linkage disequilibrium (LD) was assessed for all possible marker combinations using simulated exact tests as implemented in GENEPOP 3.3 (Raymond and Rousset 1995; Rousset 2008). A false discovery rate correction for multiple comparisons (Benjamini and Hochberg 1995) was used in tests of LD and departure from HWE. To determine whether any of the loci deviated from neutral expectations, we conducted *F*_ST_ outlier detection tests as implemented in LOSITAN (Antao et al. 2008). Any locus presenting a significantly higher or lower *F*_ST_ than predicted by neutral expectations would be considered an outlier locus, and potentially under selection. Allelic diversity, observed (*H*_*o*_), and expected heterozygosity (*H*_*e*_) were calculated at each locus for each group using GenAlEx (Peakall and Smouse 2006).

The statistical power of our dataset for testing the null hypothesis of genetic homogeneity among populations was assessed using POWSIM (Ryman and Palm 2006). The analysis was performed for four simulated populations for a range of differentiation levels (*F*_ST_ = 0.0001–0.05) using 1000 simulations and both the chi-squared and Fisher’s testing approaches. Power was estimated as the proportion of significant outcomes. Levels of genetic differentiation among groups under various grouping strategies were estimated by pairwise comparisons of *θ* (Weir and Cockerham 1984) as calculated in FSTAT (Goudet 2001). Significance was assessed at *α* = 0.05. The mean allele length was calculated for each individual at the *OtsClock1b* locus, and the group averages were compared using a Kruskal–Wallis test, implemented in R version 2.15.1 (R Core Development Team 2009).

To determine the number of discrete genetic units within the dataset without a priori grouping, the Bayesian method of Pritchard et al. (2000) was used as implemented in STRUCTURE 2.3.4. Run length was set to 500,000 MCMC replicates after a burn-in period of 100,000 using correlated allele frequencies under a straight admixture model. We varied the number of clusters (*K*) from 1 to 5 with 20 iterations per value of *K*. The most likely number of clusters was determined by plotting the log probability of the data (ln Pr(*X*|*K*)) across the range of *K* values tested and selecting the *K* where the value of ln Pr(*X*|*K*) plateaued as suggested in the STRUCTURE manual. We also calculated Δ*K* (Evanno et al. 2005) as implemented in STRUCTURE HARVESTER (Earl and vonHoldt 2012); however, due to the manner in which it is calculated, this method is incapable of inferring a *K* = 1.

Analyses of molecular variance (AMOVAs) were performed using ARLEQUIN (Excoffier and Lischer 2010) for the neutral and circadian-linked loci separately and for a range of grouping strategies including: RE/RL/SE/SR; (RE+RL)/(SE+SR); (RE+SE+SR)/RL; and RE/RL/(SE+SR). A locus-by-locus AMOVA was also run for the circadian-linked loci.

### Reproductive timing

Previously in Meadow Creek kokanee, a steep seasonal decline was reported for longevity, the period between arrival at the spawning channel and death (Morbey and Ydenberg 2003). However, longevity not only includes RLS, but also the period of waiting between arrival and nest settlement. Here, we reanalyzed 1998 and 1999 data from Morbey and Ydenberg (2003) to confirm that RLS declines with spawning date in females, using date of nest settlement as a proxy of spawning date. We also used reduced major axis (RMA) regression implemented in PROC NLP in SAS/STAT software (version 9.2; SAS Institute, Cary, NC) to estimate the slope of the seasonal decline. Compared to ordinary least squares regression, RMA regression provides a better estimate of the actual decline given uncontrolled sources of variation in the date of nest settlement (McArdle 1988).

## Results

The *Oki10* locus had nearly 50% missing data and was excluded from all analyses; the retained dataset had 2.5% missing data. None of the remaining loci displayed outlier behavior or evidence of null/false alleles. There was no evidence for LD or deviation from HWE, even prior to false discovery rate correction for multiple comparisons. Across the eight retained microsatellite loci, there was an average of 14.2 alleles per locus. Overall, *H*_*o*_ and *H*_*e*_ were 0.84 and 0.81, respectively (Table 1). The loci putatively linked to circadian functions were less variable, with 3–20 alleles each, *H*_*o*_ values ranging from 0.08 to 0.64, and *H*_*e*_ values ranging from 0.13 to 0.58 (Table 1). A single individual had an allele at *OtsClock1b* (allele 367) not previously detected in kokanee (Lemay and Russello 2014).

**Figure 2:**
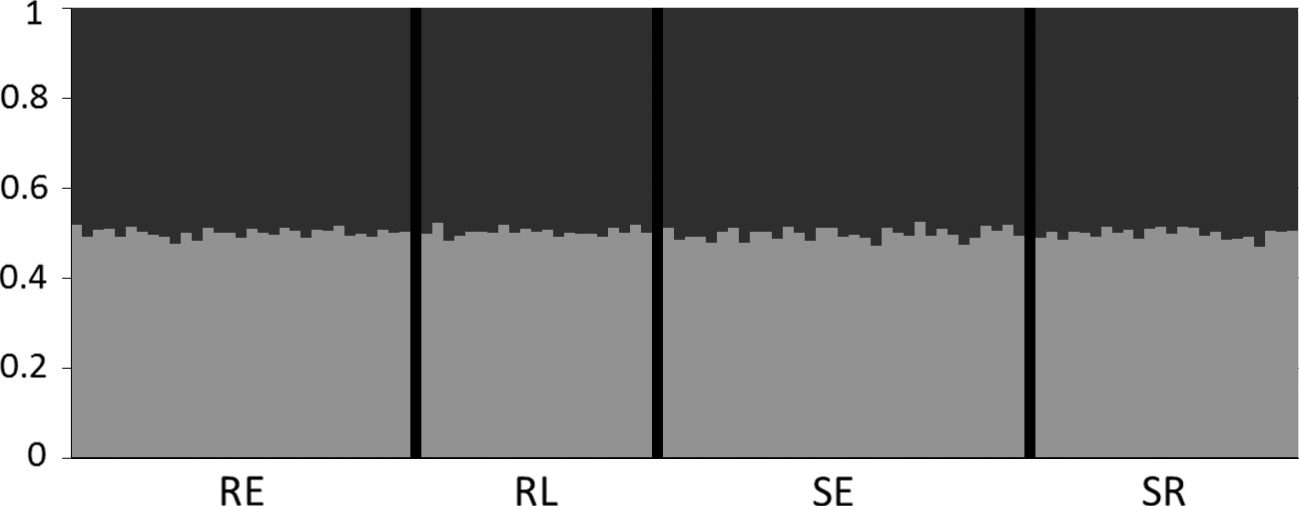
Bar plot from the Bayesian clustering analysis. The most likely value of *K* was *K* = 1, but the *K* = 2 plot is shown for display purposes. Each color represents an inferred genetic cluster. Each bar on the *x*-axis represents an individual within a group (RE = red early; RL = red late; SE = silver early; SR = silver at maturity), with the *y*-axis displaying the proportion of membership in each genetic cluster.

**Figure 3:**
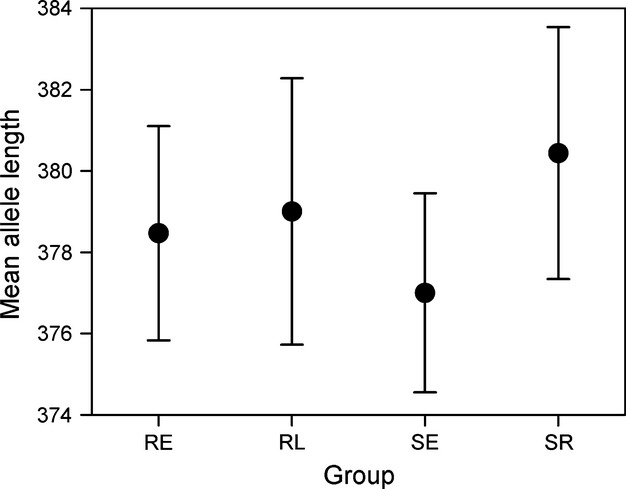
Mean allele length of individuals at *OtsClock1b* within each group of kokanee (RE = red early; RL = red late; SE = silver early; SR = silver at maturity). Bars display 95% confidence intervals.

The power analysis revealed that the recovered variation in our dataset provided sufficient statistical power (with a conventional value of 1−*β* > 0.8) to detect small levels of divergence (*F*_ST_ = 0.005), with the proportion of significant outcomes at this level equaling 0.958 and 0.934 for the chi-squared and Fisher’s exact tests, respectively (Figure S1). Despite having the power to detect even very weak differentiation, none of the pairwise comparisons of *θ* were significant among the four groups (Table S1). Likewise, >99% of the variation was distributed within populations for the AMOVAs based on the neutral and circadian-linked loci (Table 2) across all grouping strategies. Similar results were obtained in locus-by-locus AMOVAs for the circadian-linked loci analyzed separately (data not shown). The Bayesian clustering analysis indicated *K* = 1 to be the most likely (Fig. 2). The mean allele length for *OtsClock1b* also did not differ across the groups (*P* = 0.42, Fig. 3).

**Table 2:** Analysis of molecular variance (AMOVA) results showing the percentage of variation at each hierarchical level of organization for the circadian-linked and neutral microsatellite loci. The locus-by-locus analysis of the circadian loci produced highly similar results. None of the values were significant (*P* < 0.05)

Loci	Groups	Among groups	Among populations within groups	within populations
Circadian linked	RE/RL/SE/SR	–	−0.14	100.14
	(RE+RL)/(SE+SR)	−1.14	0.63	100.51
	(RE+SE+SR)/RL	−1.07	0.31	100.76
	RE/RL/(SE+SR)	−2.48	1.90	100.58
Neutral	RE/RL/SE/SR	–	−0.67	100.67
	(RE+RL)/(SE+SR)	0.19	−0.80	100.61
	(RE+SE+SR)/RL	−0.45	−0.48	110.93
	RE/RL/(SE+SR)	0.01	−0.68	100.67

### Reproductive life span

**Figure 4:**
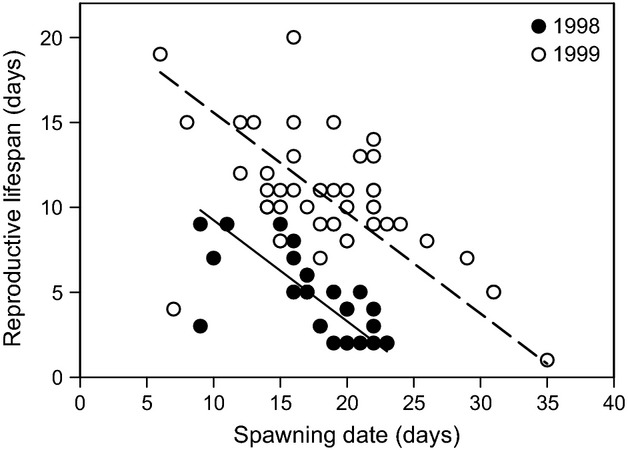
The relationship between the reproductive life span and spawning date of kokanee in 1998 (filled circles, solid line) and 1999 (open circles, dashed line). The lines show the slopes estimated from reduced major axis regression. Spawning date is the day relative to 25 August 1998 and 1 September 1999.

In 1998 and 1999, female RLS declined with spawning date (two-way ANOVA: *F*_1,75_ = 38.5, *P* < 0.0001; *β* = −0.345 ± SE = 0.056) and was longer in 1999 than in 1998 (*F*_1,75_ = 110.8, *P* < 0.0001; Fig. 4). The slope did not differ between years (*F*_1,74_ = 0.3, *P* = 0.6). Using RMA regression, the estimated slope of the decline was −0.593 in 1998 and −0.592 in 1999. Over an 18-day period, a decline of this magnitude would correspond to a total shortening of RLS by 11 days.

## Discussion

At the time scale relevant for seasonal declines in RLS, Meadow Creek kokanee did not show significant temporal structure based on genetic variation at presumably neutral loci. Among the four groups of females that differed in their arrival date (early or late) and their timing of maturation (early or late), there was no genetic differentiation (Table 2), with an overall *F*_ST_ of −0.003. Furthermore, Bayesian clustering analysis determined that there was only one panmictic group. Thus, isolation-by-time is weak at the time scale over which we observed seasonal declines in RLS. These results concerning isolation-by-time are consistent with other *Oncorhynchus* populations measured at time scales of less than 1 month (Hendry and Day 2005) and thus call into question a genetic basis for adaptive seasonal declines in RLS.

Matching the lack of isolation-by-time based on neutral genetic variation, we did not detect any population genetic structure at *OtsClock1b* or other circadian-linked loci. This result may not be surprising given only three *OtsClock1b* PolyQ length variants in our study. Moreover, no other study has reported temporal genetic structure of circadian-linked loci at such a short time scale. Only at time scales beyond a month do genetic differences begin to emerge. For example, in one of five comparisons, *OtsClock1b* showed significant divergence (*F*_*ST*_ = 0.042) between seasonal runs of Chinook salmon (*O. tshawytscha*) (O’Malley et al. 2007). Across a broad latitudinal range, genetic differences in *OtsClock1b* were found among populations of Chinook salmon, coho salmon (*O. kisutch*), pink salmon (*O. gorbuscha*), and chum salmon (*O. keta*, O’Malley and Banks 2008; O’Malley et al. 2010), as well as kokanee (Lemay and Russello 2014). Moreover, RLS have never been shown to differ between seasonal runs or to vary systematically with latitude.

Overall, phenological traits such as migration timing and maturation timing show high heritability in salmon, with median *h*^2^ = 0.51 (Carlson and Seamons 2008), and this should promote adaptation-by-time. However, empirical estimates of heritability must be treated with caution, because genetic and environmental components of phenotypic variance not only depend on the trait in question, but also on the study population and experimental conditions. In New Zealand Chinook salmon, for example, Quinn et al. (2000) tested whether phenotypic divergence in timing between introduced populations was genetically based. For each population, crosses took place in 1 day using parents with similar maturation dates. This introduces the complication of assortative mating and limits the ability to estimate heritability based on the genetic covariance of parents and offspring. In the end, they estimated very large heritabilities (>1 for migration and maturation timing), but they also observed high dispersion of timing traits among offspring of parents with similar maturation dates. In Auke Creek pink salmon, a similar design was followed except that crosses took place on two dates to target the early and late runs in this system. Similar to the Chinook salmon study, heritability was significant, but offspring within each part of the run showed high dispersion of migration timing relative to their parents (Smoker et al. 1998). Because of the heritable basis for run timing (early versus late), Kovach et al. (2012) were able to demonstrate a genetic basis underlying the trend toward earlier arrival from 1983 to 2011. Finally, Dickerson et al. (2005) and Abadía-Cardoso et al. (2013) estimated the heritability of arrival timing using parent–offspring regression. In pink salmon, Dickerson et al. (2005) estimated high heritability values for males (0.6–1.38), but values <0 for females. In steelhead (*O. mykiss*) maturing over a 2-month period, Abadía-Cardoso et al. (2013) estimated heritability to be similar between males (*h*^2^ = 0.50) and females (*h*^2^ = 0.56).

If adaptive seasonal declines in RLS are not the outcome of adaptation-by-time, what other mechanism could be responsible? An alternative explanation is condition-dependent arrival timing (Hendry et al. 1999; Hendry and Day 2005). Referred to as the parental quality hypothesis in the avian literature, condition-dependent arrival has often been used to explain seasonal declines in fitness-related traits, such as clutch size in birds. Under the parental quality hypothesis, better condition individuals arrive earlier, and reproductive investment is directly related to individual condition. This hypothesis is usually pitted against the date hypothesis, in which seasonal declines in fitness-related traits are caused by a seasonal deterioration of the environment (Verhulst and Nilsson 2008). Hendry et al. (1999) did not find broad support for condition dependence, in large part because of weak evidence for seasonal clines in body size in salmon (but see Doctor and Quinn 2009). Moreover, game theory models indicate that seasonal declines in RLS can be evolutionarily stable in the absence of condition-dependent arriving timing (Morbey and Ydenberg 2001).

Rather than breeding date flexibility, individuals may adjust their reproductive allocation in anticipation of their breeding date. Individual variation in breeding date could arise due to environmental variability or intrinsic factors (e.g., size, condition, or growth history) that affect the timing of maturation. In salmonid fishes, reproductive development seems to be controlled by developmental decisions far in advance of breeding, although the specific factors influencing variation in rate or onset of sexual maturation are not well understood (Thorpe et al. 1998; Thorpe 2007; Wright 2007). Environmental effects on the onset of reproductive development are likely to play a role in *Oncorhynchus*, given the large interannual variation in arrival timing reported in this study and others (Quinn and Adams 1996; Quinn et al. 1997, 2002; Robards and Quinn 2002; Hodgson et al. 2006). Individuals anticipating a late maturation may then favor allocation to reproductive effort over reproductive life span, thus producing a seasonal decline in RLS. This type of flexibility in response to breeding timing has been observed in birds, where manipulations of breeding date resulted in adjustments of reproductive effort (de Neve and Soler 2002; Moreno-Rueda 2004; de Neve et al. 2004). To test whether salmon can adjust their reproductive allocation in response to their maturation state at a particular time would require the induction of late- versus early-maturing individuals within a population. In a hatchery setting, subjecting a subset of individuals to different photoperiod regimes could be used to induce such variation. Pacific salmon have one chance to reproduce, and physiological flexibility in reproductive allocation could permit adaptive adjustments given the vagaries of environmentally induced variation.

One important limitation of our study is the reliance on a limited number of neutral loci to detect population structure and only three targeted gene regions to detect loci under selection. The possibility remains that a full genomic scan would reveal loci under selection in association with early- versus late-arriving or with early- versus late-maturing fish. Russello et al. (2012) applied this approach to differentiate shore- versus river-spawning ecotypes of kokanee within Okanagan Lake, British Columbia, and found 8 outlier loci associated with ecotypes despite no differentiation based on neutral loci. Although kokanee ecotypes in Okanagan Lake do differ in spawning timing, they also vary in a host of other traits (Taylor et al. 2000; Winans et al. 2003) that may be associated with the identified outlier loci (Russello et al. 2012). Demonstrating a genetic basis to the seasonal decline in RLS would provide the best evidence for the adaptation-by-time hypothesis. However, to uncover the proximate basis of seasonal declines in RLS within seasonal runs, we argue that future research effort should focus on processes operating at this scale, such as individual-level responses to phenotype, growth history, and environment.

## Data Archiving Statement

Genetic data are available from the Dryad Digital Repository: http://dx.doi.org/10.5061/dryad.98c48.

## Supporting Information

**Figure S1.** Results from a comparative power analysis implemented in POWSIM (Ryman and Palm 2006) using genotypic data for eight neutral microsatellite loci and three circadian-linked loci. **Table S1.** Pairwise differentiation (*θ*) for the four groups (RE, RL, SE, and SR) of kokanee.
