# Supplementary material for: Time scale matters: genetic analysis does not support adaptation-by-time as the mechanism for adaptive seasonal declines in kokanee reproductive life span
Source: Ecol Evol. 2014 Sep 5;4(18):3714–22. doi: 10.1002/ece3.1214 (PMC4224543; doi:10.1002/ece3.1214)
Supplement: Supplementary file 1 — Figure S1. Results from a comparative power analysis implemented in POWSIM (Ryman and Palm 2006) using genotypic data for eight neutral microsatellite loci and three circadian-linked loci. Table S1. Pairwise differentiation (θ) for the four groups (RE, RL, SE, and SR) of kokanee. [file ece30004-3714-sd1.docx]

**Supplementary Fig. 1** Results from a comparative power analysis implemented in POWSIM (Ryman & Palm, 2006) using genotypic data for eight neutral microsatellite loci and three circadian-linked loci. The analysis was performed for four simulated populations for a range of differentiation levels (*F_ST_* = 0.0001-0.05) using 1000 simulations and both the χ^2^ and Fisher testing approaches. For clarity, only the results from the χ^2^ approach are plotted, although the results using the Fisher exact test were virtually identical.

**Supplementary Table 1**. Pairwise differentiation (*θ*) for the four groups (RE, RL, SE, and SR) of kokanee. Values below the diagonal are for all 11 loci, and values above the diagonal are based on the eight neutral loci. None of the values are significant at *P* < 0.05.

|  | RE | RL | SE | SR |
| --- | --- | --- | --- | --- |
| RE | – | -0.0045 | -0.0026 | -0.0031 |
| RL | -0.0049 | – | -0.0024 | -0.0067 |
| SE | -0.003 | -0.0033 | – | -0.0031 |
| SR | -0.003 | -0.0067 | -0.0004 | – |
