# Supplementary figures and images for: Time scale matters: genetic analysis does not support adaptation-by-time as the mechanism for adaptive seasonal declines in kokanee reproductive life span
Source: Ecol Evol. 2014 Sep 5;4(18):3714–22. doi: 10.1002/ece3.1214 (PMC4224543; doi:10.1002/ece3.1214)

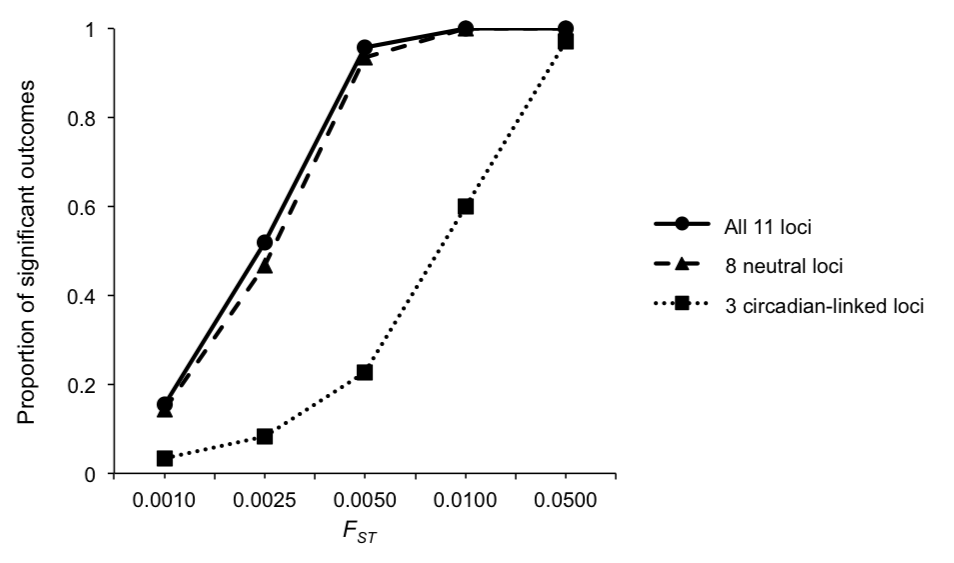

Supplement: Supplementary file 2 [file ece30004-3714-sd2.tiff]
